# Tracking the impact of COVID-19 on economic inequality at high frequency

**DOI:** 10.1371/journal.pone.0249121

**Published:** 2021-03-31

**Authors:** Oriol Aspachs, Ruben Durante, Alberto Graziano, Josep Mestres, Marta Reynal-Querol, Jose G. Montalvo

**Affiliations:** 1 Caixabank Research, Caixabank, Barcelona, Catalonia, Spain; 2 Department of Economics and Business, Universitat Pompeu Fabra (UPF), Barcelona, Catalonia, Spain; 3 ICREA, Barcelona, Catalonia, Spain; 4 Institute for Political Economy and Governance (IPEG), Barcelona, Catalonia, Spain; 5 Barcelona Graduate School of Economics (BGSE), Barcelona, Catalonia, Spain; Xiamen University, CHINA

## Abstract

Pandemics have historically had a significant impact on economic inequality. However, official inequality statistics are only available at low frequency and with considerable delay, which challenges policymakers in their objective to mitigate inequality and fine-tune public policies. We show that using data from bank records it is possible to measure economic inequality at high frequency. The approach proposed in this paper allows measuring, timely and accurately, the impact on inequality of fast-unfolding crises, like the COVID-19 pandemic. Applying this approach to data from a representative sample of over three million residents of Spain we find that, absent government intervention, inequality would have increased by almost 30% in just one month. The granularity of the data allows analyzing with great detail the sources of the increases in inequality. In the Spanish case we find that it is primarily driven by job losses and wage cuts experienced by low-wage earners. Government support, in particular extended unemployment insurance and benefits for furloughed workers, were generally effective at mitigating the increase in inequality, though less so among young people and foreign-born workers. Therefore, our approach provides knowledge on the evolution of inequality at high frequency, the effectiveness of public policies in mitigating the increase of inequality and the subgroups of the population most affected by the changes in inequality. This information is fundamental to fine-tune public policies on the wake of a fast-moving pandemic like the COVID-19.

## Introduction

The COVID-19 pandemic has had a massive impact on economic activity around the globe. To tackle the economic consequences of the pandemic, most governments have used a combination of family income support and credit facilities for firms. In particular, expanded unemployment insurance and furlough schemes have been adopted to stabilize the income of the workers, and contain the impact of the crisis on consumption and economic inequality. The concern is that a surge in inequality may erode social cohesion and spur support for populist or even undemocratic views.

Yet, how appropriate and effective these policies are remains unclear, mainly due to a lack of reliable indicators allowing to track economic activity at a fine temporal resolution. Indeed, most official statistics on inequality are available only at yearly frequency and often with long delays. This limits the ability of policymakers to rapidly adjust their responses in the effort to “flatten the recession curve” [1] after flattening the infection curve.

The COVID-19 has pushed new international initiatives to track economic activity in real time [2–6]. Researchers analyze the impact of economic stimulus packages to mitigate the effect of the COVID-19 epidemic on economic activity using high-frequency administrative data. Two examples are the effect on aggregate employment of the Paycheck Protection Program of the US [7] or the effect on consumption of the stimulus checks sent by the US Administration [8] using the data from financial aggregation and service apps [9–12].

One characteristic aspect of pandemics is their impact on inequality [13, 14]. However, official inequality measures are calculated with long lags and low frequency. In the context of a fast-moving pandemic it is important to have a high-frequency measure of inequality to evaluate the mitigating effect of policy measures. This is particularly important in countries, like Spain, that suffered very intensively the financial crisis of 2008 and that have experienced an important increase in inequality since then. This process increased the support for populist parties, which in 2008 were not represented in the parliament and in 2020 accounted for 32.8% of the representatives in Congress. It is interesting to notice that inequality increased significantly from 2008 to 2012 but the process of growing political representation of populist parties happens mostly after 2013, even though inequality was decreasing since 2013. This seems to imply that there may be a threshold level of inequality that, once overcome, can trigger a set of popular grievances that persist over time, generating increasing support for populist parties. Therefore, a further increase in inequality, even in the short run, could imply reaching a level of inequality above the threshold that triggers future tension and political unrest. It could also ignite a process of increasing support for populist parties that could easily produce a significant deterioration of the institutional stability. Ultimately, this could have a long run effect on economic performance.

This paper uses bank account data and proposes a methodology to track the impact of government policies on inequality immediately after they are taken. Inequality is a multifaceted object and can concern dimensions as different as income, wealth, education etc. Our analysis focuses on wage inequality which, in countries with a high proportion of wage-earners, is a very precise indicator of overall income inequality (as we document for Spain). We do not look at wealth inequality mainly because, using information from just one financial institution, there is a high risk of not gauging a complete picture of the financial holdings of an individual. Bank account data have many advantages to study the effect of policy responses to the COVID-19 pandemic. They provide timely and reliable information on wages and government benefits. Being able to use very granular data, and to construct a high-frequency measure of inequality, allows to tailor policies to contain the increase of inequality in general, and by subgroups of the population classified by income level, gender, age, and county of birth.

Recent research has also used bank account data to study the evolution of different macro-magnitudes at very high frequency and, in particular, the effects of the pandemic on consumption [15–17]. Our contribution to this literature is threefold. First, and opposite to many papers in this literature [9, 10], our sample is very representative of the population of Spanish wage-earners. As we show in next section, the distribution by gender and age are almost identical to the figures reported by the National Statistical Office. Second, and in contrast with a large part of the literature that uses banks accounts data, we are not analyzing the evolution of expenditure but the changes in the distribution of wages over time. Finally, the papers that have data on expenditure and income, like [16], deal with the issue of the sensitivity of consumption to income and do not consider the evolution of inequality, which is our basic objective.

We study empirically the evolution of inequality, before and after considering government support, comparing the period before the lockdown with the lockdown stage. We apply this methodology to data from a large Spanish bank. Spain is one of the countries most affected by the pandemic not only in terms of the number of people infected, but also regarding the economic impact. The comparison of the situation before and after the activation of the new policies of income support allows analyzing the effect of government interventions in the mitigation of inequality.

Using these bank account data, and our research design, we find that the largest impact of COVID-19 on inequality is transmitted through the movement of the distribution of salary changes among low wage earners. Second, we also find that most of the increase of inequality in the period after the beginning of the pandemic is mitigated by the action of the new extended unemployment benefits and furlough schemes activated by the government. There are no other changes in other government benefits during the period of analysis. We provide further details on the public income measures to support workers in the next section. Third we show that the policy response could not fully mitigate the large increase in inequality among young people and foreign-born individuals.

## Materials and methods

We study the effect of COVID-19 on inequality using bank account data from CaixaBank, the second largest Spanish bank. Caixabank had monthly records on more than 3 million wage earners in 2020, and accounted for 27.1% of the wages, salaries and benefits deposited monthly in the Spanish financial sector. In Spain, differently from other countries like the US, the payment of the salaries or benefits using checks is a very rare event. Almost all the payments of salaries and benefits use direct deposits on bank accounts.

The wages and government benefits recorded by CaixaBank provide a large, precise and granular data source. Banks’ administrative data avoid most of the problems of surveys: there are no measurement errors or imperfect recollection mistakes, and they are obtained with short delays compared to surveys. For instance, the CaixaBank data provides the universe of wages through June 15, 2020 while the latest official measure of wage inequality in Spain, produced by the National Institute of Statistics, was published at the end of June of 2020, but referred to the situation in 2018.

The granularity of CaixaBank data allows also calculating inequality for subgroups of the population. Unlike other financial institutions, such as digital banks and personal finance management software, CaixaBank collects demographic information directly (gender, age, province, country of birth). We also provide a methodology to calculate monthly Gini indices and Lorentz curves, before and after accounting for public benefits, to analyze if the schemes to support workers temporarily out of the labor market are being effective at containing inequality.

The raw data are the wages and salaries deposited monthly at CaixaBank, and they present some challenges in order to construct wage inequality measures. We restrict our sample to accounts with either only one account holder or with multiple account co-holders but only one employer paying-in wages. This way, we ensure that payrolls or transfers recorded correspond to only one individual and avoid recording multiple payrolls or transfers from multiple account holders. In addition, we exclude from the sample those individuals who died during our period of study or who did not use the bank account for their usual financial transactions during the period. Finally, to ensure some stability on the sample of individuals studied, we require observing either wages or government benefits during two months (that is, in December 2019 and in January 2020) prior to the beginning of the period of study (February 2020). The S1 File explains all the details of our methodology to select the data.

Our reference sample includes individuals aged 16-64 who received either wages or unemployment benefits in December of 2019 and January of 2020. We follow those individuals in the months starting in February 2020. Since our main source of data is related with holding a bank account it is important to start analyzing the level of financial inclusion in Spain. The data of the Global Findex, the index of financial inclusion of the World Bank, shows that 97.6% of Spanish people over 15 years old holds a bank account when the average in high income countries is 93.7%.

We exclude the self-employed from our sample since it is difficult to calculate their net monthly income from bank account data: they receive payments from many different sources, and it is complicated to calculate expenses associated with their business. However, it is important to note that the proportion of wage earners among the Spanish working population was 84.4% in the first quarter of 2020 (Labor Force Survey of Spain, EPA). The relevance of wages as the main source of income can also be seen in the similarity of the inequality measures using income or gross wages. For instance, for the last period for which both measures are available, income inequality in Spain, measured by the Gini index, was 0.345 while wage inequality was 0.343.

Since most of the individuals in the sample are workers, to analyze its representativeness in terms of the distribution of wages we compare our data with the data of the latest Spanish National Statistical Office’s Wage Survey (Encuesta de Estructura Salarial, EES). For this purpose we consider the individuals in our sample who were working in February of 2020. First, we compare the distribution of individuals by gender and age with other sources. Table 1 summarizes the comparisons. In general, samples from digital banks and financial aggregation services have more young males than the general population. This is not the case with large and diversified traditional banks like our data source, CaixaBank. Table 1 shows that the gender and age distribution of our data is very similar to the working population.

**Table 1:** Check our data versus labor surveys.

	Our sample (CBK)	EES	EPA4T19	EPA1T20
N	3,028,204	209,473	≈200,000	≈200,000
*Gender*				
Male	0.54	0.52	0.52	0.52
Female	0.46	0.48	0.48	0.48
*Age*				
≤ 19	0.01	0.00	0.01	0.01
20-29	0.18	0.12	0.15	0.14
30-39	0.25	0.31	0.25	0.24
40-49	0.28	0.30	0.30	0.30
50-59	0.21	0.21	0.23	0.24
≥ 60	0.07	0.05	0.06	0.06

*Notes*—EES stands for Encuesta de Estructura Salarial (Spanish Wages Survey); EPA4T19 refers to the sample ofemployees in the Spanish Labor Survey (EPA) in the last quarter of 2019; EPA1T2020 refers to the sample of employees in theSpanish Labor Survey in the first quarter of 2020.

In our sample, 54% of the individuals are male. This compares satisfactorily with the 52% of males in the sample of the last official survey (EES). In order to compare with more recent estimates, columns 3 and 4 include the proportions of males among employees in the Labor Force Survey of the last quarter of 2019 and first quarter of 2020. February of 2020 is between the last quarter of 2019 and the first quarter of 2020. The proportion of males is identical to the one in the EES and very close to the one in our sample. With respect to age, we also find that the proportions of workers in each age bracked in our sample are very similar to those reported in the EES and the EPA.

**Fig 1:**
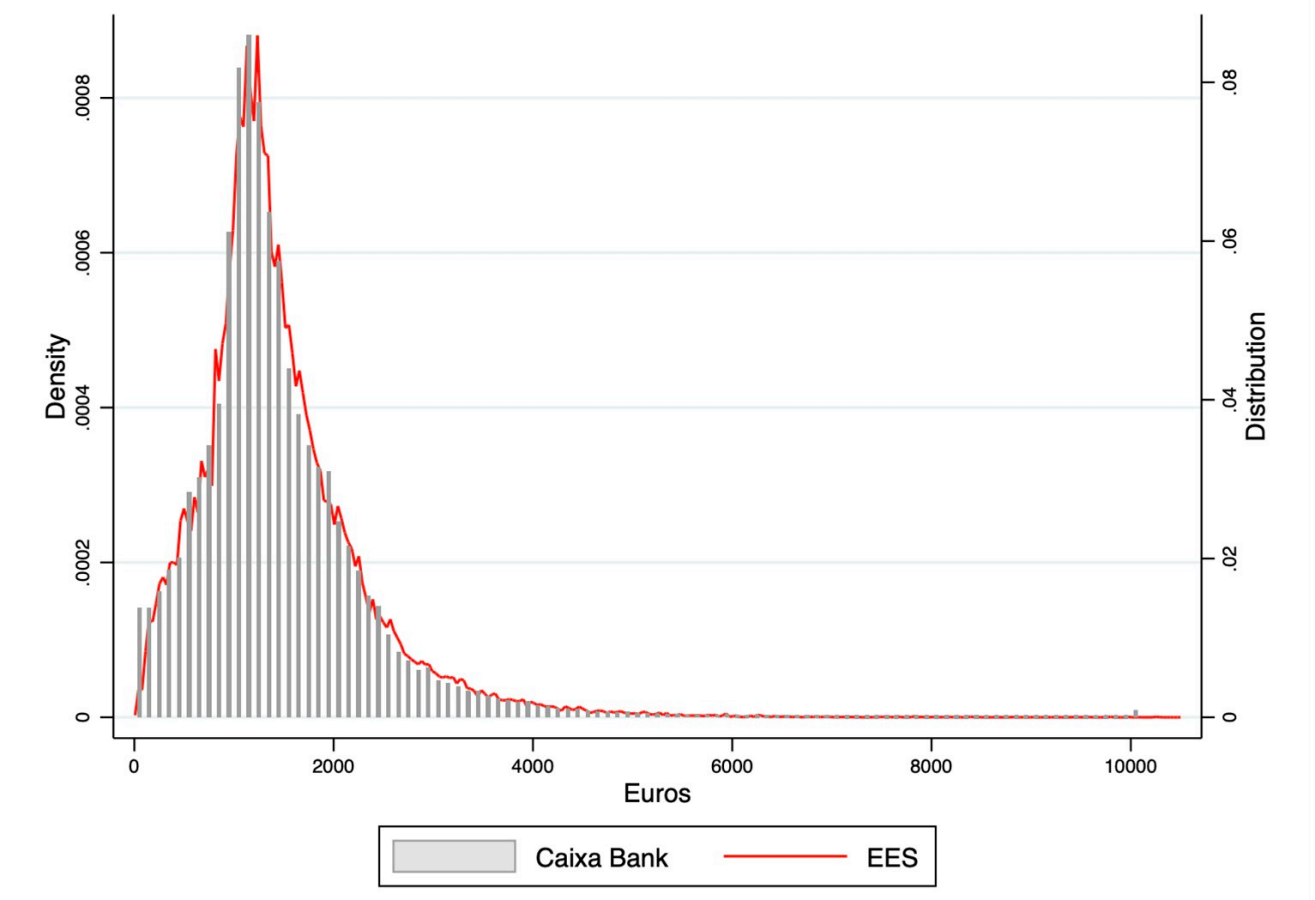
Distribution of monthly net wages: Our sample (CABK) versus the sample of the official wage survey (EES).

Fig 1 shows the distribution of the monthly wages of our sample compared with the distribution of monthly net salaries in the EES. The wages received by workers in their bank accounts are net of payroll taxes. In order to compare our data with the EES we have calculated the distribution of net salaries transforming the gross salaries of the EES into net salaries by subtracting social insurance payments and taxes withheld. The S1 File includes a detailed explanation of this transformation. Since there is a time difference between the last EES available and our data we have adjusted the wages by moving the whole distribution by the increase in the average wage since the last available EES. We can see that the histogram of the net wages of our sample is very well adjusted by the density estimation of the adjusted distribution of net salaries in the official wage survey. Both distributions are remarkably similar. The similarity of the distribution of wages, and also the characteristics of the workforce, confirms the representativeness of our sample.

Since the distributions are so similar it is not surprising to see that the quantile ratios used regularly to describe inequality are very similar in both distributions as shown in S1 Table in S1 File.

## Government support schemes for workers

The public policy response to mitigate the impact of the COVID-19 crisis in Spain has been large, as in most developed countries. For a detailed description of the economic impact of COVID-19 on the Spanish economy and the public policy reaction see [18]. The Spanish government has deployed income and liquidity support measures that are expected to reach 3.7% of GDP in discretionary measures and around 15.6% of GDP in off-budget measures [19].

Income measures to support workers have consisted mostly in the deployment of a furlough scheme (“Expediente de Regulación Temporal de Empleo”, or ERTEs) that was scarcely used until then. This scheme consists in a temporary job suspension (or a reduction in working hours) that avoids dismissals while maintaining the employment relationship. The Spanish government facilitated the use of this ERTE scheme due to COVID-19 (considering coronavirus as a force majeure, etc.) and increased coverage to all workers affected by a temporary job suspension. In addition, the benefits received did not reduce future unemployment benefit entitlements.

In addition to the job retention scheme, the government facilitated and extended the coverage of unemployment benefits. Regular unemployment benefits require a minimum of 360 days of contract employment in the previous 6 years and its duration is proportional to the amount of time worked (up to 18 months). Due to the pandemic, however, special unemployment subsidies were created for those who exhausted their unemployment benefits.

Those workers affected by job retention schemes and by unemployment received unemployment benefit transfers, which normally amounts to 70% of their social security contribution base. The schemes ensured an income stream during the duration of the contract suspension or unemployment, although of a lower amount than the regular salary.

Public transfers programs partially compensated wage losses for those workers that received them. However, despite the increase in coverage not all affected workers were entitled or had the same degree of coverage. In particular, those workers already unemployed before the pandemic or in temporary contracts that expired might not have had the right to unemployment benefits or only to reduced amounts. In addition, many of the beneficiaries experienced several months of delay before actually receiving their unemployment benefits in their bank accounts. All these developments might have affected the effectiveness of the government support to reduce inequality. This is of particular relevance in a country as Spain, which suffers from a very high labour market duality. In particular, subgroups of the population like young and foreign-born individuals are most likely to be in temporary contracts, and thus more heavily affected.

## Results

### Large effect of the shutdown on pre-benefits inequality mostly due to low wage earners

To analyze the role of government benefits on inequality our analysis considers two scenarios: pre- and post-government benefits. In the pre-benefits scenario, we consider monthly wages before taking into account the benefits. The post-benefits scenario also considers unemployment insurance benefits, subsidies and furlough schemes.

**Fig 2:**
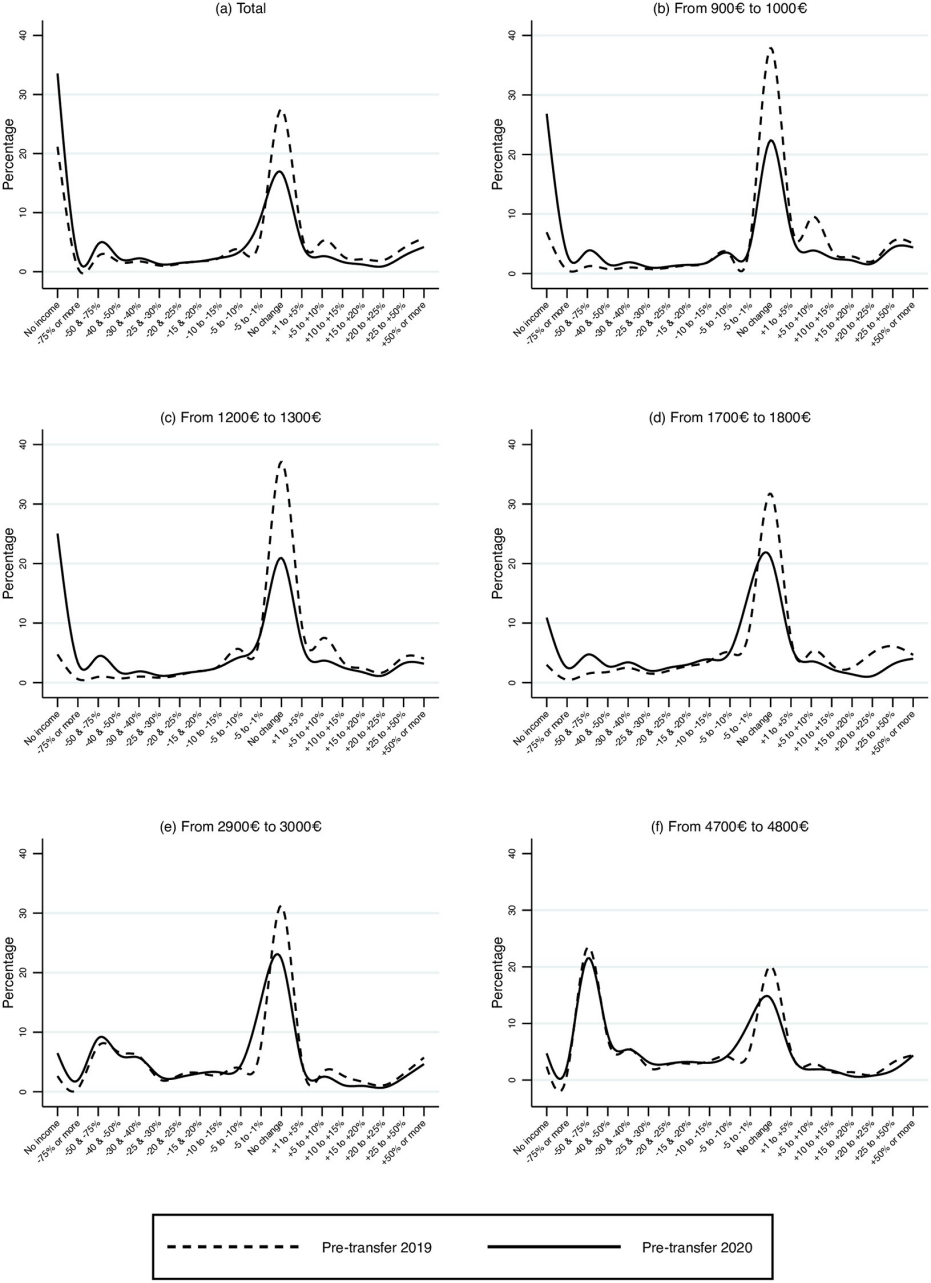
Changes in payments between April and February by level of wages in the reference period. Pre-benefits scenario. Comparing 2020 and 2019.

Fig 2 shows the distribution of changes in pre-benefit wages between February and April 2020 (i.e., before vs. during the lockdown), represented by the solid lines. The x-axis reports the percentage change in wages experienced between the two months, while the y-axis reports the share of account holders in each category. The dashed lines represent the distribution for the same months of 2019, i.e., prior to the pandemic. The top left panel reports the distribution for the entire sample; the other panels report the distribution for each of five wage brackets (measured as of February of 2020): i) the interval between 900 to 1,000 euros, which includes the 25th percentile of the wage distribution; the interval between 1,200 to 1,300 euros, which includes the median; the interval between 1,700 to 1,800 euros, which includes the 75th percentile; the interval between 2,900 and 3,000 euros, which includes the 95 percentile, and the interval between 4,700 and 4,800, which represents the top 1%.

Several interesting facts emerge from Fig 2. First, in 2020 the probability mass of the no-change interval is about half than in 2019. Compared to 2019, in 2020 a sizeable portion has moved to the no-income category. Furthermore, and most interestingly, in 2020 the probability of shifting to the no-income category is higher for individuals in the lower wage brackets.

Another noticeable aspect is that a substantial share of the highest wage earners experience a drastic wage reduction in April relative to February. This seasonal pattern, observed both in 2019 and 2020, is due to the payment of bonuses which occurs in February, and reaches over 30% for the top earners in our sample (i.e., the top 0.01%, not shown in the figure). There is no evidence of an analogous pattern for low wage earners.

S1 Fig in S1 File shows the difference in payments received by account holders between April and February after accounting for extended unemployment insurance and other benefits. Compared to the pre-benefits wages depicted in Fig 2, the shift to the no-income category is much less pronounced. There is still a large wage reduction for high earners who are largely unaffected by government transfers.

S2 Fig in S1 File compares in the same graph all the levels of initial wages, before and after government benefits, to facilitate the comparisons.

**Fig 3:**
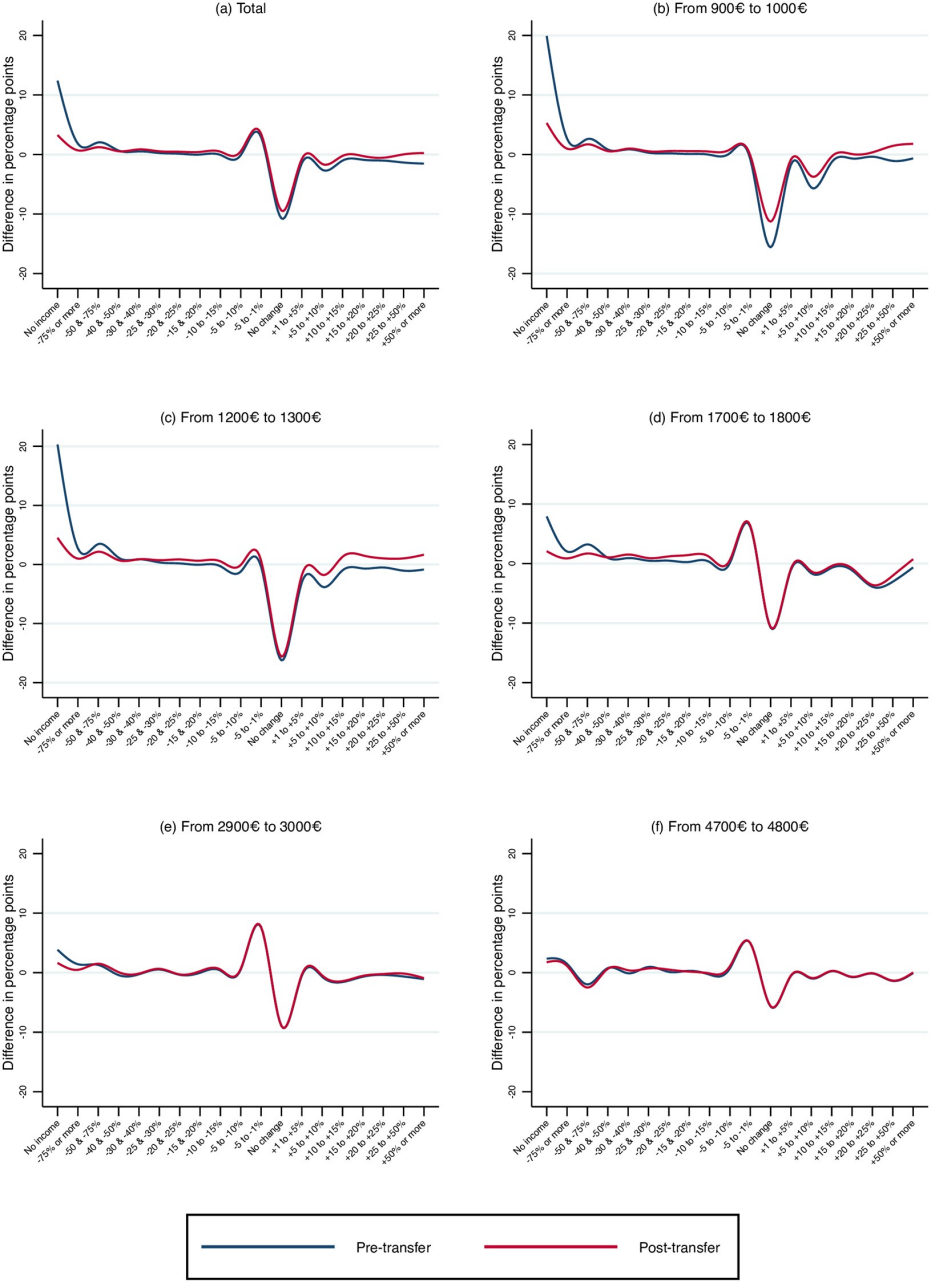
Diff-in-diffs in payments for each level of salaries in the reference month. April vs February—2020 vs 2019.

To account for seasonality Fig 3 shows the difference in the proportion of changes in salaries between April and March of 2020 net of the the difference between the same two months in 2019. The S1 File shows the precise transformation to deal with seasonality. When seasonality is controlled for, the effect of the February bonuses for high wage earners disappears. Interestingly, the effect of the pandemic on pre-transfer earnings is very different for low and high wages. For wages below 1,300 euros the lower mass in the no-change brackets is associated with a corresponding shift to no-income category. The importance of the decline of employment for the lowest-income workers is common to other countries like the US [20]. For wages above 1,700 euros, instead, the lower mass in the no-change brackets is associated with a higher share of individuals experiencing small wage cuts. S3 Fig in S1 File shows the changes for all wage categories in the same figure.

**Fig 4:**
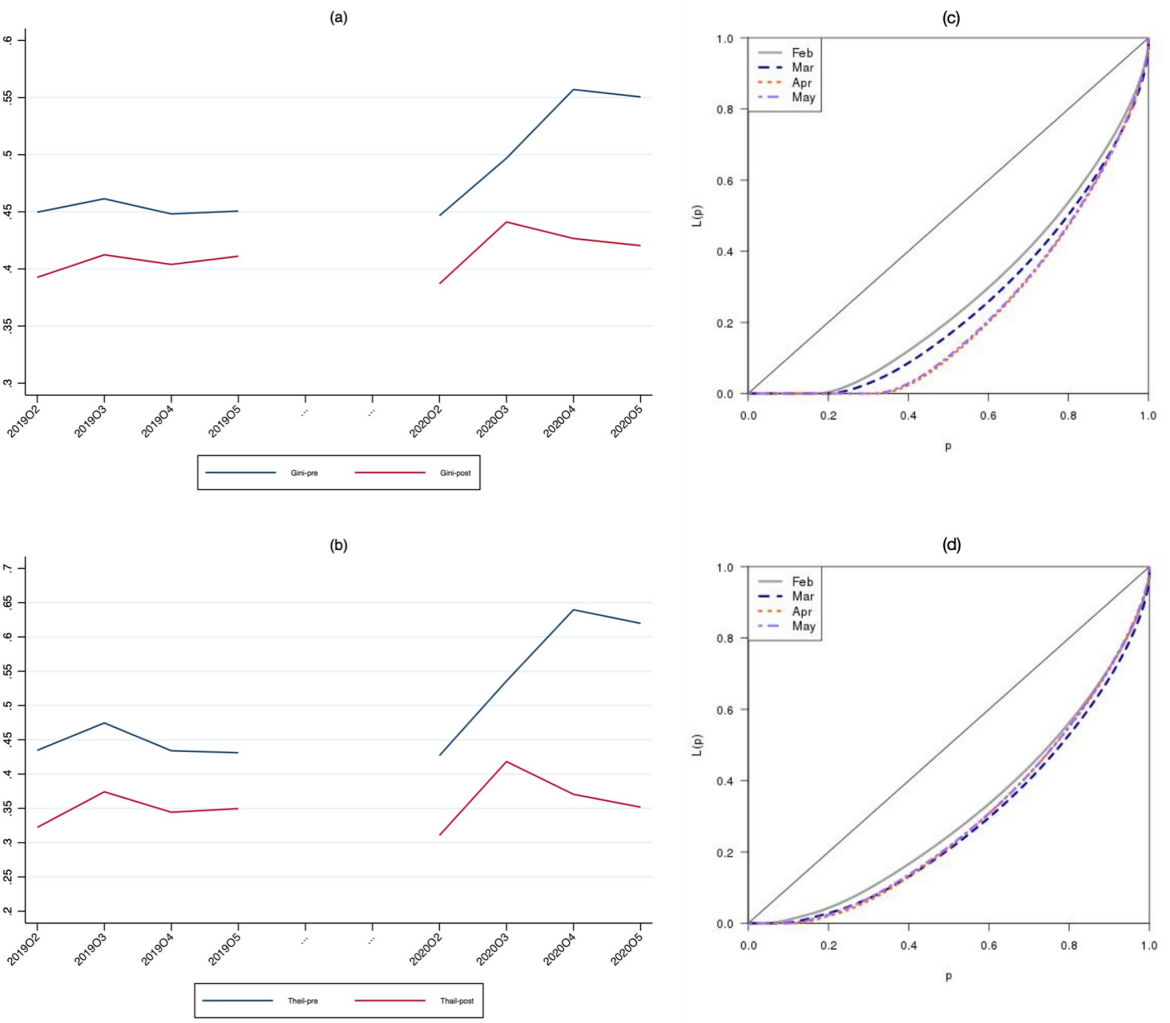
Inequality measures. (a) Gini index (b) Theil index (*α* = 1) (c) Lorentz curve: Pre-benefits, 2020 (d) Lorentz curve: Post-benefits, 2020.

To summarize the evolution of inequality we compute the Gini index. The S1 File presents a discussion of its calculation. Fig 4 Panel (a) depicts the evolution of the Gini index between February and May for 2020 and 2019, respectively. Both the pre- and post-benefits curves are basically parallel until April 2020, when the pre-benefits Gini index increases considerably while the post-benefit one only moderately. In May 2020 the pre-benefits Gini index remains very high, while the post-benefits index returns to the pre-pandemic level. From February until April of 2020 the pre-benefits Gini index increased close to 0.11 points. This implies a 25% increase in just two months. To evaluate the statistical significance of this large movement in the Gini index we can calculate the confidence intervals around our estimate. There are basically two possible procedures: using a Jakknife or a WLS estimator [21]. The S1 File describes the calculation of the standard error of the Gini index using a WLS estimator. As expected, given our large sample size, the standard error is very low (0.0002). This implies that the increase of 0.11 points observed in the Gini index between February and April of 2020 is highly statistically significant (well over the level of significance of 1%). Since the confidence intervals are tiny they cannot be visualized in the figures.

To confirm the robustness of the documented pattern to alternative measures of inequality, in Fig 4 Panel (b) we show the evolution of the Theil index, an inequality measure related to the concept of entropy and to Shannon’s index. The S1 File discusses the computation of this index. The Theil index shows a pattern very similar to the Gini index: a sizeable increase in March for both the pre- and post-benefits distribution which persists in April for the pre-benefit measure but not for the post-benefit one.

Panels (c) and (d) of Fig 4 show the changes in the pre- and post-benefits Lorenz curves respectively for every month between February and May 2020. It is apparent that, for the pre-benefits curve, the downward movement accelerates in April and stabilizes in May, while, for the post-benefit curve, the evolution is smoother.

### Within group inequality post benefits has increased among young and foreign-born people

**Fig 5:**
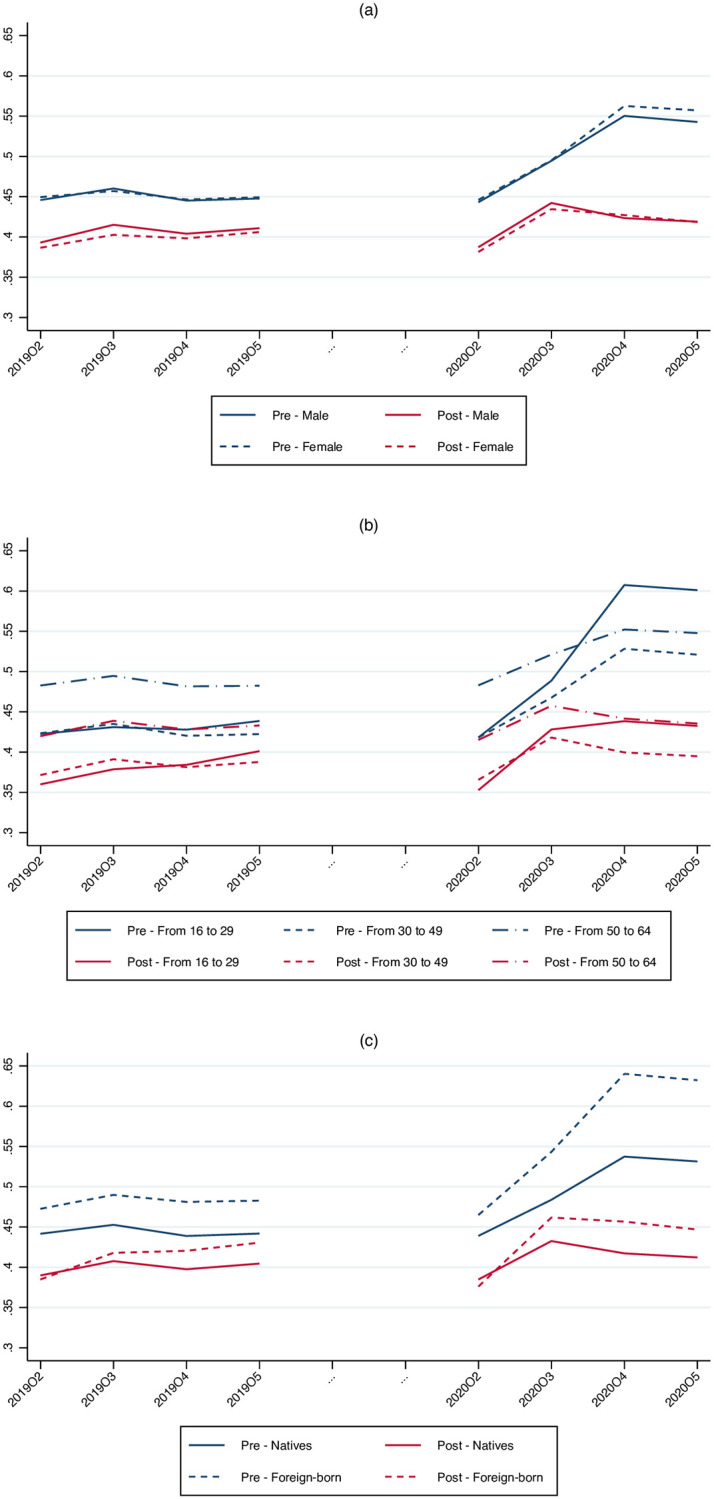
Evolution of the Gini index by gender, age and country of origin. (a) By gender. (b) By age group. (c) By place of birth.

Given the granularity of the data we can also analyze the evolution of inequality within different subgroups of the population, differentiating by gender, age, and country of origin. Panel (a) in Fig 5 shows that there are not major differences in within inequality of males and females before the shock. The magnitude of the increase in the Gini index after the beginning of the pandemic is similar across genders before public transfers, but slightly higher for females in the post-benefits case.

Panel (b) of Fig 5 shows the evolution of inequality for different age groups. For the youngest cohort (i.e., 16 to 29 years old), there is a considerable increase in the Gini index for pre-transfer earnings. The other groups also experience an increase in inequality though much smaller than for the young. The spike in the Gini index for the young is mitigated when considering the distribution of post-benefit earnings. Yet, the level of post-benefits inequality for this group is still remarkable, both in absolute and in relative terms. Such increase is arguably related to the fact that young workers account for a high proportion of temporary jobs in low wage occupations.

Panel (c) of Fig 5 shows the evolution of the Gini index separately for foreign-born individuals and for natives. As of January 1st 2020, foreign-born individuals represented 14.77% of the total Spanish resident population. Looking at the distribution of pre-benefits earnings, it is clear that inequality increases much more among foreign born than among natives. Such increase is less pronounced when looking at the post-benefits distribution, though, in this case as well, the Gini index for foreign born is significantly higher than for natives.

Interesting differences emerge when dividing foreign-born individuals by the per capita GDP level of the country of origin. For example, as shown in S4 Fig in S1 File, while post-benefits inequality decreases over time for both natives and foreign-born from high-income countries, it remains high for foreign-born from low-income countries.

The disproportionate increase in post-benefit inequality among poorer migrants attests to their vulnerability in times of crisis as their social welfare net is thinner. Foreign born workers from low income countries tend to have occupations with low salaries, and a high proportion of temporary jobs. In many cases they work without a formal contract which means that they cannot prove they were working before the pandemic and, therefore, they cannot get the benefits that other workers get. On the other hand expatriates from high income countries still enjoy a high salary.

Finally, inequality increases more in regions that rely heavily on tourism (e.g., Balearic and Canary Islands) than in other parts of the country (S5 Fig in S1 File). This is not surprising since the touristic sector is characterized by a high proportion of low wage workers who, as shown above, are the ones most affected by the job losses and wage cuts caused by the pandemic.

## Discussion

The financial crisis of 2008 generated a large increase in inequality in many countries. When some countries were still trying to recover from the financial crisis a new shock, the COVID-19, has hit the economy. Recent research shows that social distancing laws are not responsible for the economic harm [15] and the responses to emergency declarations are strongly differentiated by income [22]. In this paper we show that the economic impact is also very heterogeneous by income level which, in turn, is reflected in large increases in inequality before governments policy response.

Our findings contribute to a recent literature on the measurement of economic indicators in real-time, or at very high frequency. Most of the economic research on the impact of COVID-19 has concentrated on its effect on consumption [6, 8, 15–17]. We present evidence on the impact of COVID-19 on economic inequality. Our findings show that, before accounting for extended unemployment insurance and furlough benefits, the economic impact of the pandemic caused a large increase of inequality. After considering public benefits the effect of the crisis on inequality is mitigated. We show how bank account data of a representative financial institution can be used to track inequality and monitor the effect of economic polity on its evolution. In contrast with some previous research that uses data on personal finance websites and bank accounts, our data replicates very precisely the distribution of the population of wage earners.

We present evidence that shows a very heterogeneous impact of the pandemic on inequality by income level, age and country of birth of the individuals. Our methodology could be applied to many other countries that have introduced income-support schemes similar to the ones considered in Spain (furlough benefits and extended unemployment insurance). Tracking, at high frequency, the effect of policy responses on inequality allows tuning the policy instruments to mitigate inequality, targeting the groups that contribute the most to the increase of inequality.

## Supporting information

S1 File (PDF)
