# Supplementary material for: Tracking the impact of COVID-19 on economic inequality at high frequency
Source: PLoS One. 2021 Mar 31;16(3):e0249121. doi: 10.1371/journal.pone.0249121 (PMC8012053; doi:10.1371/journal.pone.0249121)
Supplement: S1 File — (PDF) [file pone.0249121.s001.pdf]

S1 File

Materials and methods

Sample and data

Our inequality indicators are constructed using de-identified data obtained from CaixaBank, a private bank. All the individual data have been anonymized, and there is not a single individual who could be traced in the data. All data processing has been conducted by authorized CaixaBank personnel following the bank's strict data privacy guidelines. Researchers outside of the organization (CaixaBank) did not have direct access to any individual data. Only aggregated measures and indices were available for non-CaixaBank researchers. The sample contains a panel of more than 3,000,000 wage earners and benefits recipients who represent around 27% of all the wage earners of Spain. It covers 10 months spanning during the first five months of 2019 and 2020. The raw information includes the dates of the payments, the amount, the type of payment, the number of account holders, and the demographic characteristics of the individuals (gender, age, province of residence and place of birth). To turn the raw data into a dataset useful for research on inequality we need to deal with some challenges. We restrict the universe of account holders in several ways:

- To ensure that payrolls or transfers recorded correspond to only one individual, and avoid recording multiple payrolls or transfers from multiple account holders, we consider accounts with either only one account holder or with multiple account co-holders but only one employer paying-in wages. Around 5% of individual bank accounts had multiple holders receiving two or more payrolls or subsidies and were excluded from the sample.

- We exclude from the sample those individuals who died during our period of study or who did not use the bank account for their usual financial transactions during the period. In particular, we identify each month those clients who are actively using their bank account and perform at least two non-automatic transactions (payments, etc) during the last two months. The resulting attrition rate is only around 0.7%.

- To ensure some stability on the sample of individuals studied, we require observing either wages or public benefits during two months (that is, in December 2019 and in January 2020) prior to the beginning of the period of study (February 2020). This type of stability conditions is standard when using banks or personal finance management apps data.

Therefore, the sample considers individuals aged 16-64 who received either wages or government benefits in December of 2019 and January of 2020. We follow those individuals in the months starting in February 2020. Salaries in month t are defined as the sum of all wage payments received in a particular bank account from the 16th of that month until the 15th of the following month. In Spain, employees' payments are usually deposited monthly, towards the end of the month, but wages received in any other frequency (weekly, fortnightly, etc) are also captured. Extended unemployment insurance benefits paid in month t cover the same time period but are paid in the first 10-12 days after the beginning of the month and, in particular, include those workers in the new furlough schemes.

Methods

The wages received by workers in their bank accounts are net of payroll taxes (tax withholds and Social Security contribution). In order to compare our data with the

official EES we have calculated the distribution of net salaries transforming the gross salaries (GW) of the EES into net salaries (NW) as

$$GW = BW + OC + B \tag{1}$$

$$NW = GW - SS - Taxes \tag{2}$$

where BW is the base salary, OC is the overtime compensation; B is the bonus or any other extraordinary payments, SS are Social Security contributions and taxes are withheld taxes.

In Figs S1 and S2 we calculate the distribution of the change in salaries between April (period of shutdown of the economy) and February of 2020 (before the beginning of the shutdown). Therefore, our basic transformation is

$$\Delta y_{k,A-F} = \frac{NW_{kA} - NW_{kF}}{NW_{kF}} \tag{3}$$

$$F_{k,2020}(d-1,d) = F_{k,2020}[d-1 < \Delta y_{k,A-F} < d] \tag{4}$$

$$\tag{5}$$

where $\Delta y_{k,A-F}$ is the increase in net wages from February to April $(A-F)$ for the quartile k of salaries and $F(.,.)$ is the distribution of changes in net wages that is included in the interval $d-1$ to d (for instance, from 1% to 5%). Since there is seasonality we calculate the difference between the distribution in 2020 and the distribution in 2019

$$\Delta F_k(d-1,d) = F_{k,2020}(d-1,d) - F_{k,2019}(d-1,d) \tag{6}$$

These differences are shown in Figs S3.

We use several indices to measure inequality. The basic and standard indicator of inequality is the Gini index.

$$G = \frac{1}{2N^2\bar{y}} \sum_{j=1}^{m} \sum_{i=1}^{m} n_j n_i |y_j - y_i| \tag{7}$$

where N is the number of people, m is the number of income classes, n_j is the number of individuals in class j, $\bar{y}$ is the mean of income, and y_i is income class i. The Generalized entropy family is defined as

$$GE(\alpha) = \frac{1}{N\alpha(\alpha - 1)} \sum_{i=1}^{N} \left[\left(\frac{y_i}{\bar{y}} \right)^{\alpha} - 1 \right] \; for \, \alpha \neq 0, 1 \tag{8}$$

where α defines each of the functions in the family. If $\alpha = 1$ then we obtain the Theil index,

$$GE(1) = T = \frac{1}{N} \sum \left(\frac{y_i}{\bar{y}} \right) ln \left(\frac{y_i}{\bar{y}} \right) \tag{9}$$

We calculate the confidence interval for the Gini index to show the statistical significance of the indices reported in the text. There are two basic procedure to perform this calculation: using a Jackknife or a Weighted Least Squares (WLS) estimator. It is well known thta both procedure produce the same estimators for large sample size which is our case[21]. The WLS estimator is calculated by running the regression

$$i = \theta + u_i \tag{10}$$

where u_i is a heteroskedatic error with variance equal to σ^2/y_i. This implies that the

previous regression can be transformed into a regression with an homoskedastic error,

$$\sqrt{y_i}i = \theta\sqrt{y_i} + \epsilon_i \tag{11}$$

Therefore, the standard error of the Gini index is

$$std(Gini) = \frac{2std(\hat{\theta})}{N} \tag{12}$$

where N is the number of observations.

Tables and figures

S1 Table: **Inequality: basic percentile ratios (net wages)**

	Our sample (CBK)	EES 2014
P90/P10	4.24	4.12
P90/P50	1.88	1.87
P10/P50	0.44	0.45
P75/P25	1.85	1.83

Notes - EES stands for Encuesta de Estructura Salarial (Spanish Wage Survey).

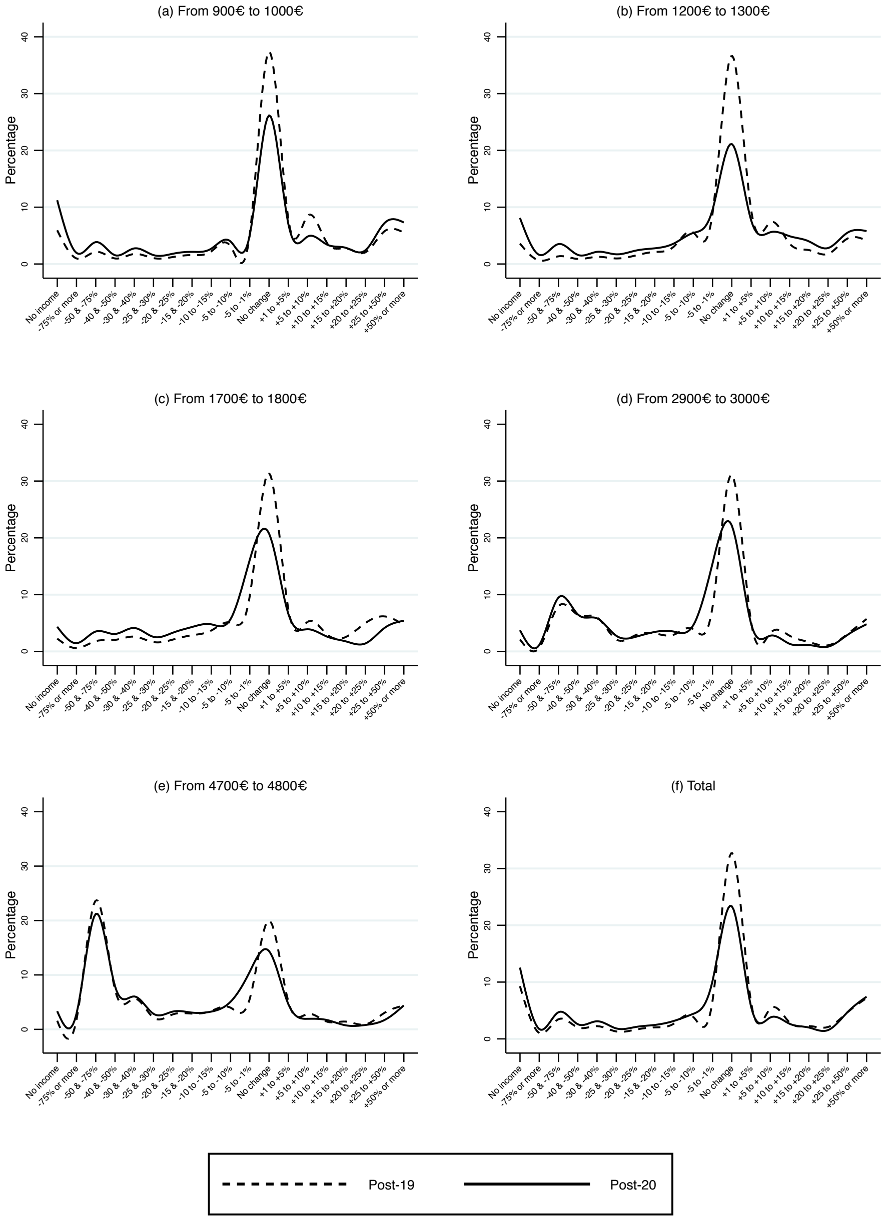

S1 Fig**: Changes in payments between April and February by level of wages in the reference period. Post-benefits scenario. Comparing 2020 and 2019**

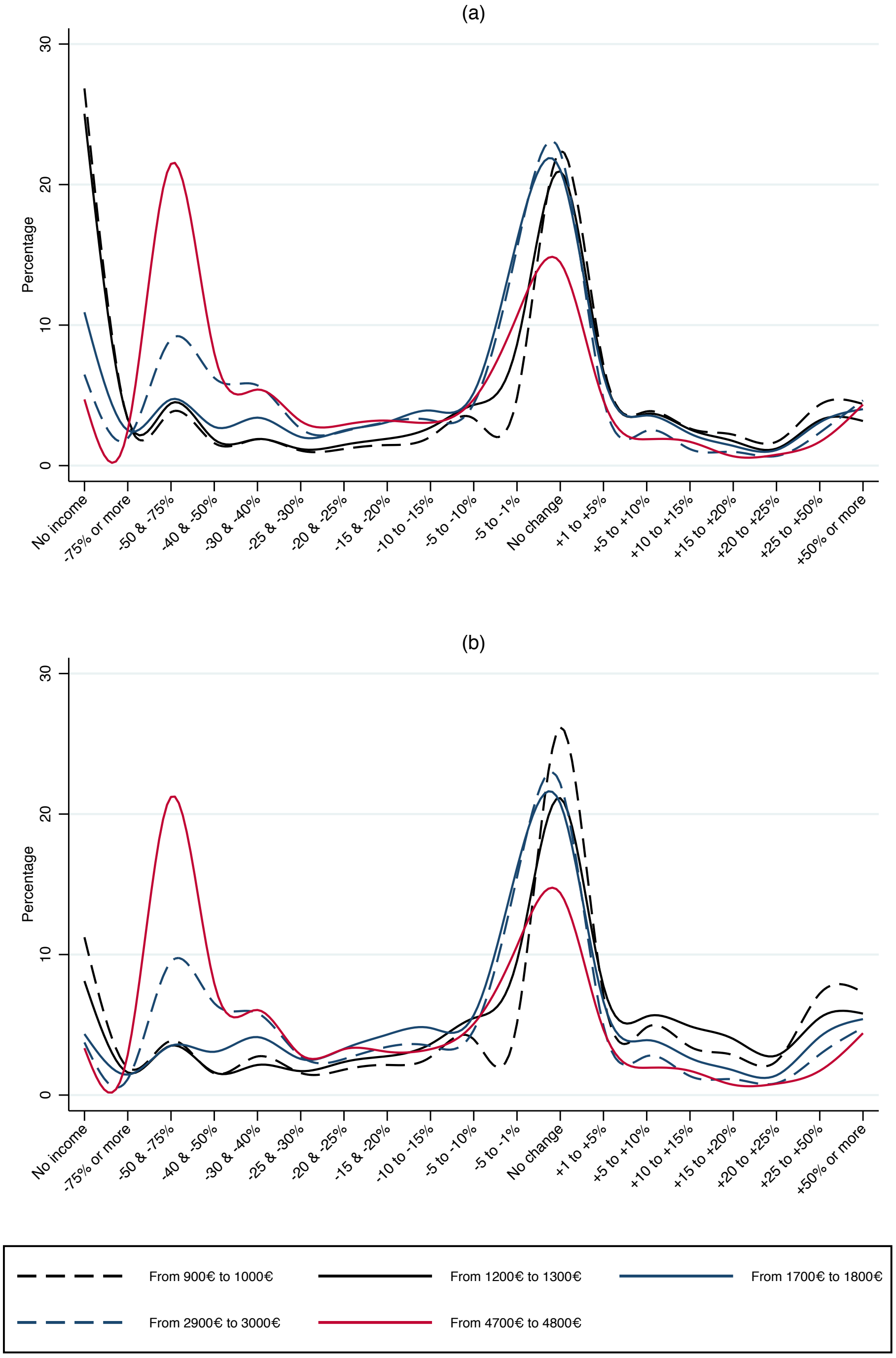

S2 Fig: Change in payments received by workers between April 2020 and February 2020
(a) Pre-benefits. (b) Post-benefits

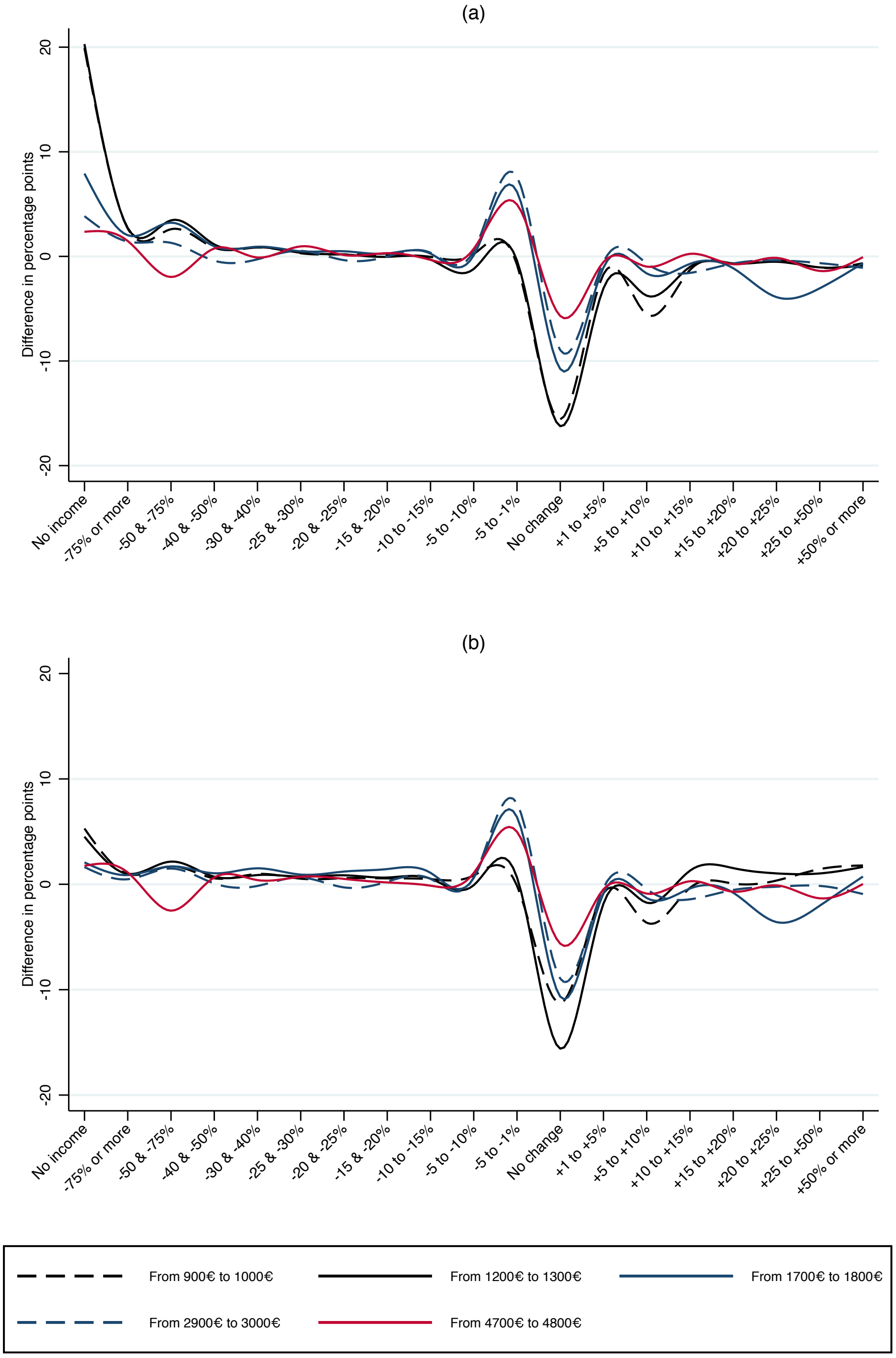

S3 Fig**: Diff-in diffs in payments received by workers: April vs February comparing 2020 vs 2019**
(a) Pre-benefits. (b) Post-benefits

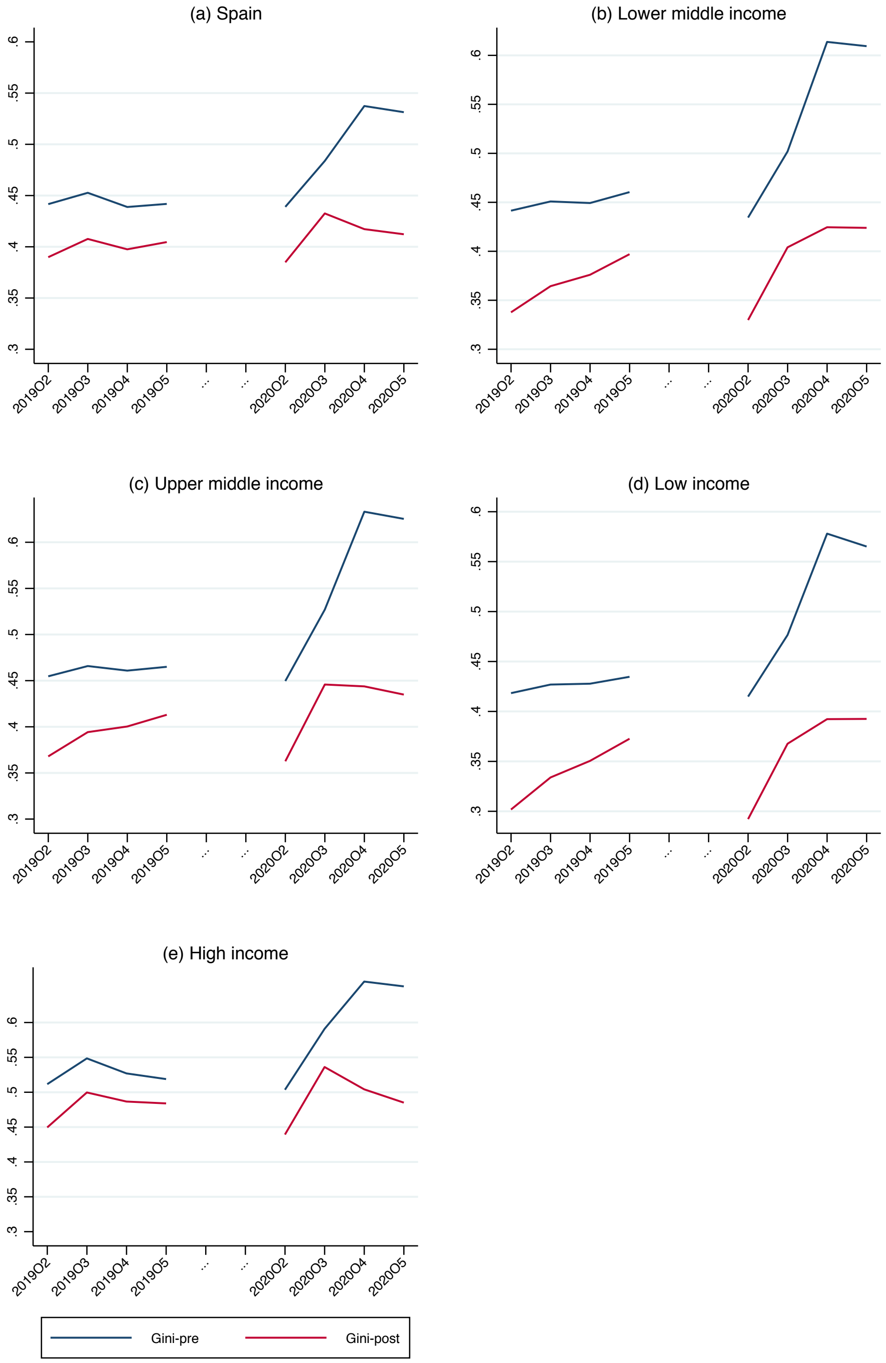

S4 Fig: **Evolution of the Gini coefficient by country of origin**
(a) All (b) Lower middle income (c) Upper middle income (d) Low income (e) High income

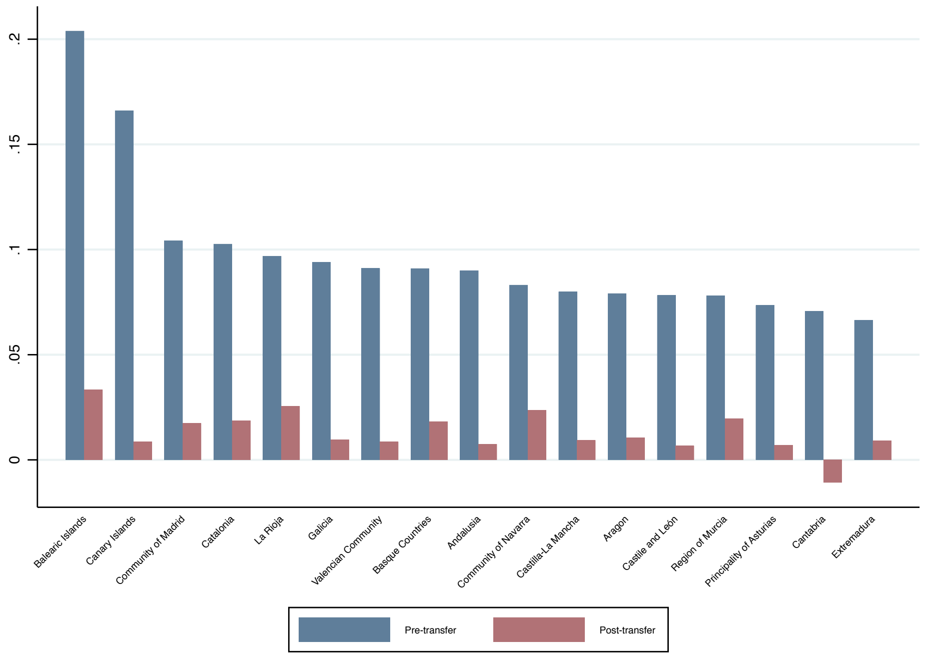

S5 Fig: Changes in the Gini coefficient by region
